# Goal-Directed Mechanical Ventilation: Are We Aiming at the Right Goals? A Proposal for an Alternative Approach Aiming at Optimal Lung Compliance, Guided by Esophageal Pressure in Acute Respiratory Failure

**DOI:** 10.1155/2012/597932

**Published:** 2012-09-17

**Authors:** Arie Soroksky, Antonio Esquinas

**Affiliations:** ^1^Intensive Care Unit, E. Wolfson Medical Center, Halohamim st 62, 58100 Holon, Israel; ^2^Sackler Faculty of Medicine, University of Tel Aviv, 69978 Tel Aviv, Israel; ^3^Intensive Care Unit, Hospital Morales Meseguer, Avenida Marqués de Los Velez s/n, 30500 Murcia, Spain

## Abstract

Patients with acute respiratory failure and decreased respiratory system compliance due to ARDS frequently present a formidable challenge. These patients are often subjected to high inspiratory pressure, and in severe cases in order to improve oxygenation and preserve life, we may need to resort to unconventional measures. The currently accepted ARDSNet guidelines are characterized by a generalized approach in which an algorithm for PEEP application and limited plateau pressure are applied to all mechanically ventilated patients. These guidelines do not make any distinction between patients, who may have different chest wall mechanics with diverse pathologies and different mechanical properties of their respiratory system. The ability of assessing pleural pressure by measuring esophageal pressure allows us to partition the respiratory system into its main components of lungs and chest wall. Thus, identifying the dominant factor affecting respiratory system may better direct and optimize mechanical ventilation. Instead of limiting inspiratory pressure by plateau pressure, PEEP and inspiratory pressure adjustment would be individualized specifically for each patient's lung compliance as indicated by transpulmonary pressure. The main goal of this approach is to specifically target transpulmonary pressure instead of plateau pressure, and therefore achieve the best lung compliance with the least transpulmonary pressure possible.

## 1. Introduction

Patients with severe respiratory failure exhibiting decreased respiratory system compliance with hypoxemia or carbon dioxide retention are often difficult to ventilate and or oxygenate with current guidelines that limit applied plateau pressure. Furthermore, applying mechanical ventilation while limiting plateau pressure without assessment of respiratory system mechanics may result in application of inappropriate positive end expiratory pressure (PEEP) and inspiratory pressures. 

Thus, while these guidelines recommend a certain limit of plateau pressure, they do not take into consideration chest wall mechanics, which can only be assessed by partitioning respiratory system into its components by esophageal balloon and assessment of pleural pressure.

Without partitioning of the respiratory system into its components, one cannot ascertain and identify the factors contributing to low respiratory system compliance.

Identifying the dominant factor affecting respiratory system compliance by measuring transpulmonary pressure may better direct and optimize mechanical ventilation. Thus, instead of limiting mechanical ventilation by plateau pressure, PEEP and Inspiratory pressure adjustment would be individualized specifically for each patient's lung compliance as indicated by transpulmonary pressure. 

The main goal of this approach is to specifically target and achieve best possible lung compliance by assessment of transpulmonary pressure instead of plateau pressure and adjust PEEP according to chest wall and lung compliance instead of total respiratory system compliance.

## 2. The Validity of Esophageal Balloon as a ****Surrogate of Pleural Pressure

Historically, esophageal balloon has been used for several decades to estimate pleural pressure. The assumption that esophageal pressure reflects pleural pressure is based on the notion that pressure in the adjacent pleura is transmitted to the esophagus [1]. This is supported by several historical studies demonstrating reasonable correlation between pleural and esophageal pressures [2–4]. However, pressure within the pleural space is not uniform. The pressure in the dependent and basal regions close to the diaphragm is greater than in the upper regions of the thoracic cage. This nonuniform pleural pressure in the upright patient exerts nonuniform pressure on the esophagus as well. Thus, in the upright position pressure measured within the esophagus varies according to the level or position of the catheter within the esophagus [5]. However, in the supine critically ill and mechanically ventilated patient pressure in the pleural space distributes differently than in the upright position. It is thought that in the supine position esophageal pressure is higher than in the upright position with resulting decreased lung compliance [5, 6]. The increase in pleural pressure in supine position is mainly caused by mediastinal structure weight that distributes differently than in the prone position. The mid third of the esophagus is thought to be the most representative and reflect most closely the effective pleural pressure [7].

Effective pleural pressure is the pressure that results from actual flow and pressure applied to the respiratory system, and thought to represent the combined effects of the different pressures found in different regions of the pleural space. Thus, although effective pleural pressure is not as accurate as pressure measurement of a specific region within the pleural space, in the clinical scenario, it does give us a reasonable clinical approximation of the average pleural pressure.

Consequently, measurement of esophageal pressure may be used as a rough estimate of pleural pressure. However, such an inference bears with its limitations, which have to be taken into account when interpreting measurements of esophageal pressure. These include the fact that in the clinical setup of critically ill patient who is mechanically ventilated and therefore in the supine or semirecumbent position, the weight of mediastinal structures such as the heart has to be accounted for. In a report by Washko et al. [5], postural changes on esophageal pressure measurements were studied on 10 healthy subjects. They showed that mediastinal structures added 3 ± 2 cm H_2_O to the measured esophageal pressure. However, it should be noted that with increasing airway pressure, there is a possibility for a concomitant decrease of superimposed pressure [8]. This could partly be explained by a possible shift of blood out of the thorax with increasing airway and pleural pressure. 

Thus, the appropriate correction factor that should be applied when we interpret esophageal pressure measurements is still controversial. Nevertheless, Talmor et al. used a similar correction in two recent reports [9, 10]. They subtracted 3 cm H_2_O for the possible weight of the heart, and another 2 cm H_2_O to correct for the effects of air volume within the esophageal balloon catheter. Thus, in their studies, in order to better approximate pleural pressure, 5 cm H_2_O was subtracted from the measured esophageal pressure. 

Weight from mediastinal structures is not the only factor that may influence accurate estimation of pleural pressure. Other factors may affect esophageal pressure measurements. These include muscle contraction that can affect intrathoracic pressures in a regional way depending on the groups of muscles that are active [11], catheter position within the esophagus [2], active tension in the walls of the esophagus [12], uneven distribution of pleural surface pressure, and esophageal spasm or contraction.

## 3. Clinical Interpretation of Esophageal Balloon Measurements

**Figure 1:**
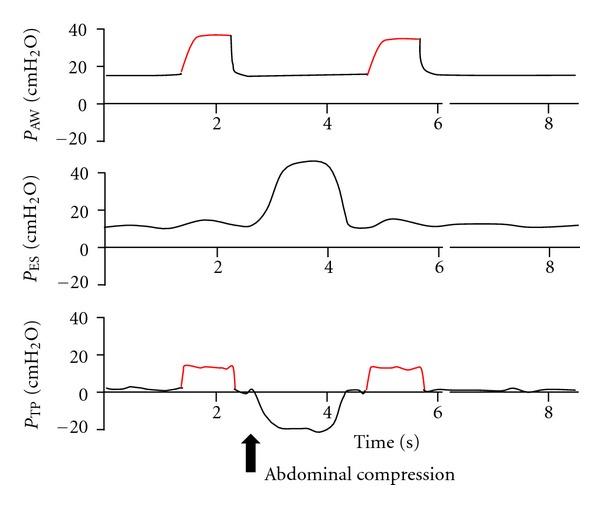
Pressure tracing of esophageal balloon with its tip in the stomach (60 to 70 cm below the incisors). Black arrow indicates gentle compression of the abdomen by the examiner. Catheter position in the stomach is also indicated by the smooth nature of the pressure tracing of the esophageal balloon and the lack of the effect of heart beat on the pressure tracing. *P*_Aw_ = airway opening pressure, *P*_ES_ = esophageal pressure, *P*_TP_ = transpulmonary pressure.

The proper interpretation of esophageal balloon measurements begins with correct esophageal catheter insertion. We usually insert the catheter well beyond 40 to 50 cm below the incisors. The purpose of advancing the catheter beyond 50 cm below the incisors is to assure that catheter tip is well within the stomach. This can be ascertained by gentle compression of the abdomen and inspection of esophageal pressure waveform (Figure 1). 

Once a positive inflection of esophageal pressure waveform is noticed with abdominal compression, the catheter is then gently withdrawn cephalad until cardiac pulsations, sometimes “saw tooth” like in appearance, simultaneous with heart beat can be noticed on the pressure tracing. This usually happens when the catheter is right adjacent to the heart, and its tip is about 40 cm from the incisors. At this level, the esophageal balloon is usually located at mid to lower third of the esophagus. Further confirmation of proper positioning of esophageal balloon can be obtained by performing the occlusion test [7, 13], in which the patient makes inspiratory or expiratory effort during airway occlusion and observing similar changes in esophageal and airway pressures.

However, occlusion test cannot be performed on paralyzed patients, in such cases for verification of correct catheter position, we may need to relay only on waveform pressure tracing interpretation. 

After verifying a correct esophageal balloon placement, measuring esophageal pressure at end expiration and end inspiration is most informative and allows us to partition respiratory system into its components. 

**Figure 2:**
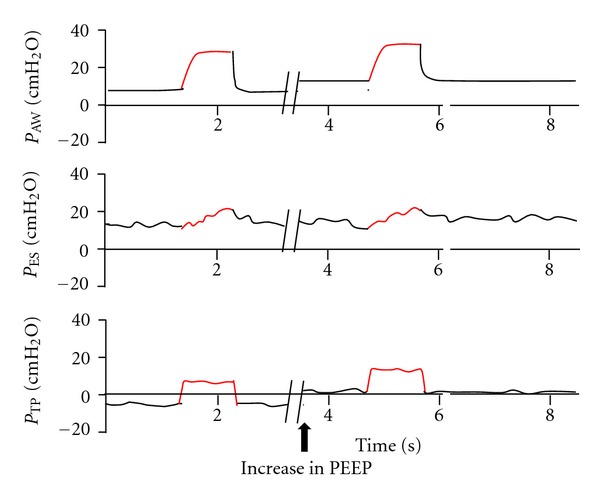
Negative transpulmonary pressure at end-expiration may subject the lungs to cyclic collapse. Black arrow indicates the point where PEEP was raised to a level that would ensure a slightly positive end-expiratory transpulmonary pressure. *P*_Aw_ = airway opening pressure, *P*_ES_ = esophageal pressure, *P*_TP_ = transpulmonary pressure.

The value measured at end expiration is calculated by subtracting end-expiratory esophageal pressure (EEPes) from airway pressure (Pao). It is mostly influenced by the applied external PEEP and by the chest wall effect. Ideally, this value should be slightly positive. A negative value indicates that the applied PEEP is actually lower than pleural pressure. This may be associated with cyclic alveolar lung units that collapse at end expiration. Thus, adjustment of PEEP to ensure positive end expiratory pressure may prevent the damage associated with the shearing forces of cyclic inflation and deflation (Figure 2).

Furthermore, PEEP adjustment guided by esophageal pressure measurements allows us to fine-tune it to ensure that the applied PEEP be at least in the magnitude of the estimated pleural pressure, while at the same time avoiding a too high value that could cause over stretching. 

Similarly, transpulmonary pressure (PL) measured at end inflation reflects the actual distending pressure acting on the lungs. Normally, it should not exceed 20 to 25 cm H_2_O. 

In patients with high pleural pressure a low PL can usually be found. This can easily be appreciated by the following equation.

(1) $$\begin{array}{c} P_{\text{PL}}=P_{\text{AW}}-\frac{E_{\text{CW}}}{E_{\text{TOT}}}, \end{array}$$

where as *P*_PL_ is Pleural pressure, *P*_AW_ is the airway opening pressure, and *E*_CW_ and *E*_TOT_ are chest wall elastance and total elastance, respectively. As an example, in a patient with high PIP of 30 cm H_2_O, without measuring pleural pressure, one may assume that the lungs are subjected to over distension [14, 15]. However, suppose that upon measurement of esophageal pressure, a pleural pressure of 20 cm H_2_O is found, in such a case there would be a low transpulmonary pressure of 10 cm H_2_O. Theoretically, in such a patient, identifying a high pleural pressure as the cause of the high PIP gives us the option to increase the limit of PIP beyond the traditional plateau pressure of 30 cm H_2_O. By doing so, even though the traditional PIP upper limit of 30 cm H_2_O is exceeded, the lungs are still subjected to a distending pressure that is well within the safe and accepted limits of the recommended transpulmonary pressure. This practice however should be exercised with caution, and the recommended 6 ml/kg of tidal volume should not be exceeded.

**Figure 3:**
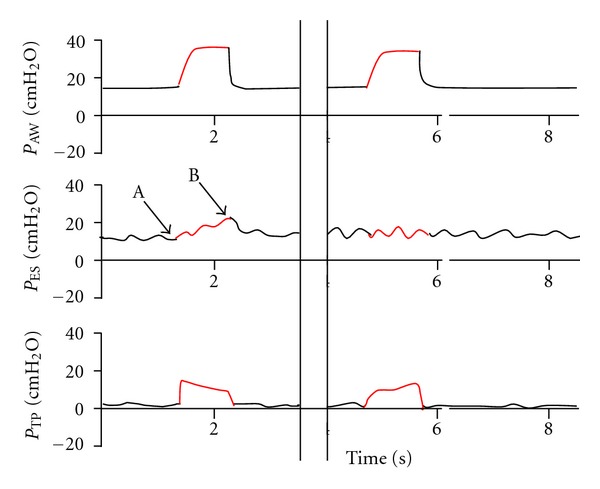
The left part of the pressure tracing shows a compliant lung that transmits part of the applied airway pressure to the pleura. The difference in pressure between points A and B represents the actual pressure transmitted to the pleura. The right part of the pressure tracing demonstrates a noncompliant lung that transmits little or no pressure to the pleura. *P*_Aw_ = airway opening pressure, *P*_ES_ = esophageal pressure, *P*_TP_ = transpulmonary pressure.

Analyzing the shape of the esophageal pressure tracing may provide additional information. In a patient with “stiff lung” airway pressure is only partially transmitted to the pleura, and in severe cases may not be transmitted at all (Figure 3). This is in opposite of very compliant lungs where a clear difference between end-expiratory and end-inspiratory pressure can be observed. This pressure difference is the actual pressure that is transmitted to the pleura (Figure 3). 

**Figure 4:**
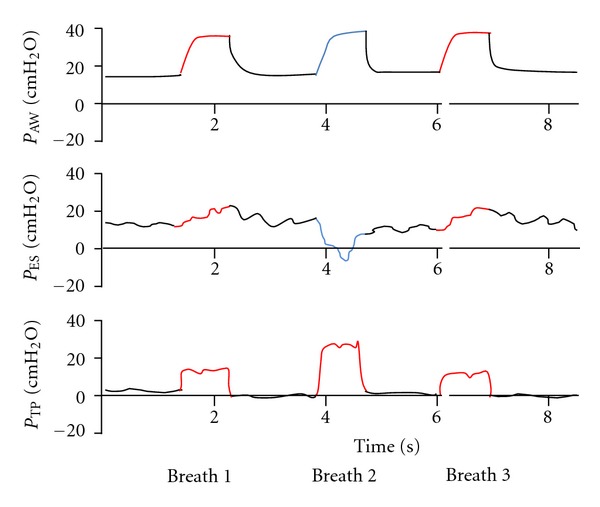
Pressure tracing demonstrating mandatory breaths delivered with inspiratory pressure of 20 cm H_2_O (breaths number 1 and 3). The second breath is initiated by the patient and is assisted with pressure support of 20 cm H_2_O by the ventilator. The large inspiratory effort by the patient (breath 2) results in a negative deflection on the esophageal pressure tracing. This negative deflection generates high transpulmonary pressure, in this example close to 30 cm H_2_O. *P*_Aw_ = airway opening pressure, *P*_ES_ = esophageal pressure, *P*_TP_ = transpulmonary pressure.

An interesting phenomenon that we have observed during the routine use of esophageal catheters is high transpulmonary pressures during patient-initiated spontaneous breath (Figure 4). A large inspiratory effort that initiates assisted pressure support delivery may result in large transpulmonary pressure. As there is no, or only limited patient effort during mandatory breath, this phenomenon is not observed during ventilator-initiated mandatory breath. The same phenomenon was recently described by Yoshida et al. [16], in an experimental model of acute lung injury in rabbits. Similarly to our observation, Yoshida and his colleagues point to a combination of mandatory breath superimposed on a strong spontaneous breathing effort which in spite delivery of tidal volume results in high transpulmonary pressure, which may promote lung damage. 

## 4. Clinical Evidence for the Use of Esophageal Balloon in Mechanically Ventilated Patients

The use of esophageal balloon in the clinical setup has not been described extensively in mechanically ventilated patients. Historically, its use has been cumbersome, not always reproducible, not widely available, and therefore, it was mainly used for research purposes. 

Thus, it is only in the last six years that studies were published reporting mainly case series of patients whose mechanical ventilation was guided by esophageal balloon pressure measurements with assessment of pleural pressure. In the first large series, Talmor and his colleagues [10] described the feasibility of esophageal balloon catheter use in seventy patients with acute respiratory failure of all causes. 

The decision to insert esophageal balloon was based on clinical grounds, and there were no systematic selection criteria. In this case series, Pes at end-expiration averaged 17.5 ± 5.7 cm H_2_O and 21.2 ± 7.7 cm H_2_O at end-inflation. Interestingly, there was no clear association between these measured esophageal pressures and body mass index or chest wall elastance. Estimated transpulmonary pressure (PL) was positive in most patients and was 1.5 ± 6.3 cm H_2_O at end-expiration, and 21.4 ± 9.3 cm H_2_O at end-inflation. Interestingly, PL at end-expiration was significantly correlated with PEEP. However, only 24% of the variance in PL was explained by airway opening pressure (*P*_ao_), and 52% was due to variation in *P*_es_. These findings demonstrated the significance of chest wall as a major factor contributing to low respiratory system compliance. Two years later, Talmor et al. reported on another trial [9]. In this study, patients with ARDS were randomly assigned to be mechanically ventilated with PEEP adjustments guided by esophageal pressure, or according to ARDS Net recommendations (control group). The primary endpoint was improvement in oxygenation. The secondary endpoints were respiratory system compliance and patient outcomes. The study was stopped prematurely by the safety committee due to significantly large differences in oxygenation between the two groups, after recruiting only 61 patients. The PaO_2_ : FiO_2_ at 72 hours was 88 mm Hg higher in patients treated with mechanical ventilation with esophageal balloons than in control patients (95% confidence interval, 78.1 to 98.3; *P* = 0.002). Similarly, respiratory system compliance was improved and was significantly better in the esophageal pressure-guided group at 24, 48, and 72 hours. This improvement can largely be attributed to the generally higher values of PEEP applied in the esophageal pressure-guided group (17 ± 6 versus 10 ± 4, *P* < 0.001).

Furthermore, It should be noted that by 72 hours, transpulmonary end expiratory pressure was negative in the control group (0.1 ± 2.6 versus −2.0 ± 4.7, *P* < 0.06), whereas in the esophageal pressure guided group, this value was positive, this was achieved by significant increase in PEEP. Mortality, a secondary outcome measure, had a trend toward a better outcome in the esophageal pressure guided group, but it did not reach statistical significance. 

Recently Grasso et al. [17] described 14 patients with severe ARDS due to influenza who were referred to their center for possible treatment with ECMO. Upon measurements of esophageal pressure, half of the patients were found to have high transpulmonary pressure (27.2 ± 1.2 cm H_2_O), and they were all treated with ECMO. 

However, the other half of the patients were found to have a low end-inspiratory transpulmonary pressure (16.6 ± 2.9 cm H_2_O), thus allowing the increase of PEEP with improvement in respiratory parameters (improved oxygenation index from 37.4 ± 3.7 to 16.5 ± 1.4, *P* = 0.0001) and eventually the successful management of these patients conservatively without ECMO. This example only demonstrates how knowledge of pleural pressure may actually change patient treatment. 

The main limitation of all these reports is the primary outcome, which is mostly the rate of improvement in oxygenation and lung compliance. By now, we already know that improvement in oxygenation, a main outcome measure in these studies, is not necessarily associated with improved patient survival. Although in the study of Talmor et al. [9], there was a trend towards improved survival in the esophageal pressure guided group, probably due to small sample size, it did not reach statistical significance. Thus, for a study to achieve a statistical significance for a major outcome such as improved survival, at least a few hundred patients, all monitored with esophageal balloon, have to be recruited. Such an immense effort can only be achieved with an international multiple center study. However, since the use of esophageal balloon is still not a common practice in many ICUs, there is no forecast for such an effort in the near future. 

## 5. Are We Aiming at the Right Goals? Should We Change Our Practice? 

In the last two decades, significant progress has been made in our understanding of the implications of inappropriate mechanical ventilation. Patient exposure to high inspiratory pressures and high tidal volumes is now recognized as major risk factors for lung damage. In addition, application of inappropriately low or high PEEP values may also contribute to inappropriate ventilation with worsening hypoxemia and increase in shunt fraction.

The recognition that limiting inspiratory pressure may decrease mortality has led to the development of lung protective ventilation. Although this approach may offer a mortality benefit in the general population of mechanically ventilated patients, it does not address individual patients lung and chest wall mechanics. Therefore, treating individual patients with a generalized approach ignores variations between patients and does not take into account different patient lung and chest wall mechanics. 

Based on the few reports published in the last few years [9, 10, 17], we have adopted an alternative approach, whereby in our ICU, an esophageal balloon is routinely being used in patients with severe respiratory failure for assessment of pleural pressure. The use of esophageal balloon has been further promoted by the recent availability of commercially available esophageal nasogastric catheters, some of which are even equipped with an internal lumen that allows simultaneous nasogastric feeding. 

In their study, Talmor et al. [9] reported that in approximately one-third of the patients, the balloon could not be passed into the stomach, and esophageal placement was confirmed only by the presence of a cardiac artifact on the pressure tracing. However, with the use of recently commercially available nasogastric esophageal balloon catheters which are thicker, less pliable, and with internal lumen for nasogastric feeding, we are now able to use esophageal pressure monitoring for prolonged periods while at the same time continue feeding these patients. 

There is a paucity of information on when to use esophageal balloon and on what patients. The existing reports did not use clear inclusion criteria. Therefore, there are no clear definitions or recommendations. However, our group has developed systematic clinical criteria that could guide patient selection for esophageal balloon insertion. We do not use esophageal balloon in every patient who is mechanically ventilated. Instead, we use esophageal balloon only in the most severe cases of respiratory failure. 

To be eligible for esophageal balloon insertion, a prerequisite of high-peak inspiratory pressure (plateau pressure of 25 to 30 cm H_2_O) has to be present, and at least one of the following four severity criteria has to be met.

1. Low total respiratory system compliance (*C*_*T*_), defined as less than 40 ml/cm H_2_O.
2. P/F ratio of less than 300.
3. Need for a PEEP greater than 10 cm H_2_O to maintain SaO_2_ of >90%.
4. PCO_2_ over 60 mm Hg, or PH less than 7.2 that is attributed to respiratory acidosis.

With the selection of appropriate patients, we may see a continued spread and increased use of esophageal balloon in the near future. Together with the already available published evidence indicating a beneficial effect of esophageal balloon pressure measurements on oxygenation and lung compliance raise important questions.

- Are the Acute Respiratory Distress Syndrome Network (ARDSNet) recommendations [30] appropriate for all patients?
- Should we continue and ignore large variations in lung and chest wall mechanics in individual patient?
- Furthermore, while we mechanically ventilate patients with severe respiratory failure, are we aiming at the right goals? In other words, is limiting plateau pressure to 30 cm H_2_O without taking into account lung and chest wall mechanics is a good and sound physiological practice?

The answer to all these questions is probably no. 

With regard to ARDSNet recommendations, many feel that they are reasonable recommendations for a general population of mechanically ventilated patients who are not the most severe ones.  Table 1 summarizes the studies on mechanical ventilation strategies in ARDS patients in the last decade. 

Table 2 summarizes the few meta-analysis examining ventilation strategies in ARDS. Among the many studies examining the most appropriate PEEP in ARDS patients several stand out in terms of sample size and quality. For example, these studies [31–33] investigated the effects of higher PEEP values in ARDS patients and failed to show improved survival. A common finding to these studies is the improvement in outcome measures such as oxygenation, hospital stay, and perhaps length of mechanical ventilation. However, survival, a major outcome in these studies, was not affected by higher PEEP values. 

Conversely, in patients with severe ARDS, the recommendations of ARDSNet may result in under treatment in terms of applied PEEP which according to the ARDSNet algorithm in these patients may be too low. This was shown in a meta-analysis of three randomized studies [34]. Treatment with higher versus lower levels of PEEP was not associated with improved hospital survival. However, a subgroup analysis on patients with severe ARDS defined by P/F ratio <200 did show improved survival in patients treated with higher PEEP values, with mortality of 34.1% versus 39.1% (adjusted RR, 0.90; 95% CI, 0.81–1.00; *P* = .049). Thus, patients with milder acute lung injury (paO_2_/FiO_2_ ratio > 200) treated with higher PEEP had a trend toward harm. 

This suggests that patients with low or normal pleural pressure who are exposed to higher PEEP values, as a result, may be subjected to higher transpulmonary pressures with possible lung over inflation, that may promote ventilator-induced lung injury, barotraumas, or decreased cardiac output. 

Since pleural pressure was not assessed in these studies, there is a possibility that part of the beneficial effect of raising PEEP was due to offsetting a negative transpulmonary pressure at end expiration (EXPtp). This possibility is supported by previous reports [9, 10] demonstrating improved oxygenation and lung compliance when end-expiratory transpulmonary pressure was kept positive. The common belief is that by keeping EXPtp positive, we may prevent cycling collapse with deflations at end expiration, and thus derecruitment of alveolar lung units at end expiration. 

Therefore, a recommendation suggesting a common PEEP value to all patients may result in over inflation in patients with low pleural pressure, and at the other end of the spectrum may ignore and miss those patients with high pleural pressure due to chest wall effect. These may be the patients with abdominal surgery, obese patients, and patients with severe chest wall edema. Common to all these patients is a phenomena where abdominal content pushes the diaphragm cephalad while encroaching upon the lungs. This results in increased pleural pressure due to chest wall effect and is usually associated with low transpulmonary pressure. 

Thus, while these patients demonstrate high-peak Inspiratory pressures (PIPs) with decreased total respiratory system compliance. The actual transpulmonary pressure is low, and usually well below the upper limit of 25 cm H_2_O. 

In fact, in the study of Talmor et al. [9], the average end inspiratory transpulmonary pressure (EIPtp) was 8.6 ± 5.4 in the conventional treatment group, and 7.9 ± 6.0 in the esophageal pressure guided group. These relatively low EIPtp values suggest that in at least some patients with high PIP, the dominant component responsible for the high PIP is the high pleural pressure. Thus, knowledge of lung mechanics allows us to raise PEEP and inspiratory pressure. Therefore, instead of aiming at a plateau pressure of 30 cmH_2_O, we are now able to target specifically lung compliance. By adopting this approach, Talmor et al. [9] raised PEEP without necessarily increasing significantly PIP. In their study, the average plateau pressure in the esophageal-guided group was not significantly greater than in the conventional group. This effect was probably achieved by increasing PEEP which resulted in improved lung compliance. 

Lastly, are we aiming at the right goals? Should a plateau pressure of 30 cm H_2_O be our limit? Based on the few reports available, the answer is probably no, or at least not for everybody. 

Based on these reports and on our experience, we believe that many mechanically ventilated patients will be able to be managed by the standard ARDSNet approach. These patients usually exhibit acceptable total respiratory system compliance and reasonable oxygenation, thus negating the need for high PEEP which in this group of patients is not beneficial [34]. However, a significant proportion of patients may present with severe ARDS characterized by low total respiratory system compliance and with high PIPs. These patients should be suspected to have a chest wall effect. 

However, without partitioning of the respiratory system into its components by measuring esophageal pressure with assessment of pleural pressure we may not appreciate the true contribution of chest wall on lung mechanics. In such patients, without identifying the factors contributing to low respiratory system compliance, we may apply inappropriately low levels of PEEP. 

While considering the approach of esophageal pressure monitoring for guiding mechanical ventilation, some limitations of this approach should be familiar and acknowledged. Firstly, it should be noted that the few available reports that evaluated PEEP adjustments based on esophageal pressure were performed mostly on patient with lung lesions that are more diffuse in their nature, such as ARDS or bilateral pneumonia. Therefore, in patients with pulmonary lesions involving one lung, such as one-sided pneumonia, the effects of adjustment of PEEP based on esophageal pressure in these patients are still unknown. Secondly, as with any other instance when high PEEP values are applied, the hemodynamic side effect of exposing patients to high positive pressure should be kept in mind and hemodynamic compromise should be identified promptly, especially in hypovolemic patient. 

In conclusion, we believe that the standard ARDSNet approach will suffice for most of the mildly ventilated patient. In these patients, a plateau pressure limit of 30 cm H_2_O is an acceptable limit. However, in patients with severe ARDS and low compliance, esophageal balloon and bedside assessment of pleural pressure should be routinely used when available. This approach allows us to partition the respiratory system into its components allowing us to apply the most appropriate PEEP and inspiratory pressure for each individual patient. Thus, with this individual patient approach, we are aiming at a target that results in best lung compliance. And instead a plateau pressure, our limits should be end inspiratory transpulmonary pressure that is lower than 25 cm H_2_O, while at the same time keeping a slightly positive end expiratory transpulmonary pressure.

Finally, the last major advancement in the field of mechanical ventilation was based on physiologic research performed from many committed investigators who provided the pathophysiologic knowledge of ventilator-induced lung injury. The interpretation of these studies has led trials such as the ARDSNet published in 2000, and eventually to the development of lung protective ventilation approach.

We hope that the next major leap in mechanical ventilation will be an international effort similar in size to the ARDSNet that would compare in patients with severe ARDS, the general approach of ARDSNet with an approach that adjusts mechanical ventilation individually, tailored to each patient as guided by esophageal balloon measurements.

## Figures and Tables

**Table 1:** Randomized controlled trials of ARDS ventilation strategies. (last 10 year-humans) years 2000–2012.

Author/year/ref	Mechanical ventilation strategy	Study aims	Major observations
Hodgson et al., 2011, [18]	Recruitment PEEP and PMV	Open-lung strategy titrated PEEP and targeted and low airway pressures	Open-lung strategy was associated with greater amelioration in some systemic cytokines, improved oxygenation, and lung compliance over seven days.

Chung et al., 2010, [19]	HFPV	HFPV and low tidal volume ventilation	Acidosis and hypercapnia induced by VT reduction and increase in PEEP at constant *P* (plat) were associated with impaired right ventricular function and hemodynamics despite positive effects on oxygenation and alveolar recruitment.

Mekontso Dessap et al., 2009, [20]	Sighs superimposed on lung PMV	Impact of acute hypercapnia and augmented positive	Sighs superimposed on lung-protective mechanical ventilation with optimal PEEP improved oxygenation and static compliance in patients with early ALI/ARDS.

Badet et al., 2009, [21]	Recruitment maneuvers on lung PMV	Comparison of optimal PEEP and recruitment maneuvers, lung-protective mechanical ventilation	Sighs superimposed on lung-protective mechanical ventilation with optimal PEEP improved oxygenation and static compliance.

Mercat et al., 2008, [22]	Recruitment maneuvers	PEEP strategy for setting PEEP	Increasing alveolar recruitment while limiting hyperinflation did not significantly reduce mortality. However, it did improve lung function and reduced the duration of mechanical ventilation and duration of organ failure.

Meade et al., 2008, [23]	PMV with low VT	Strategy using low tidal volumes, recruitment maneuvers	Open lung resulted in no significant difference in all-cause hospital mortality and high PEEP or barotrauma compared with an established low-tidal-volume protocoled ventilation strategy.

Wolthuis et al., 2008, [24]	Low VT and PMV	Lower Tv and PEEP prevent pulmonary inflammation in patients without preexisting ALI	Lower VT and PEEP may limit pulmonary inflammation.

Pachl et al., 2006, [25]	HFOV	Normocapneic HFOV affects differently extra pulmonary and pulmonary forms of ARDS	HFOV recruits and thus it is more effective in ARDS.

ALI: acute lung injury, ARDS: acute respiratory distress syndrome, HFOV: High-frequency oscillatory ventilation, HFPV: high-frequency pulmonary ventilation, *P* (plat): Plateau pressure, PEEP: positive end expiratory pressure, PMV: protective mechanical ventilation, and VT: tidal volume.

**Table 2:** Meta-analysis studies of ARDS ventilation and strategies (last year 10 year-humans) years 2000–2012.

Author/year/ref	ARDS-Mechanical ventilation strategies	Major results	Study limitations	Recommendations
Burns et al., 2011 [26] Petrucci and Iacovelli, 2007 [27]	Pressure and volume limited ventilation	PVL strategies reduce mortality. Mortality is significantly reduced at day 28 and at the end of hospital stay. Increment of paralytic agents.	Clinical heterogeneity, such as different lengths of follow-up and higher plateau pressure in control arms in two trials, make the interpretation of the combined results difficult.	There was insufficient evidence concerning morbidity and long term outcomes.
Putensen et al., 2009 [28]	Low VT strategy and outcomes	Available evidence from a limited number of RCTs shows better outcomes with routine use of low VT but not high PEEP ventilation in unselected patients with ARDS or acute lung injury.	limited number of RCTs	Best outcomes with routine use of low VT but not with high PEEP.
Hodgson et al., 2009 [29].	Recruitment maneuvers	Recruitment maneuvers significantly increased oxygenation above baseline levels for a short period of time in four of the five studies that measured oxygenation.	There were insufficient data on length of ventilation or hospital stay to pool results.	There is no evidence to make conclusions on whether recruitment maneuvers reduce mortality or length of ventilation in patients with ALI or ARDS.

ARDS: adult respiratory distress syndrome, PVL: pressure volume limited. VT: tidal volume.
